# Exploring the Role of miR-132 in Rat Bladders and Human Urothelial Cells during Wound Healing

**DOI:** 10.3390/ijms252011039

**Published:** 2024-10-14

**Authors:** Clara I. Chamorro, Magdalena Fossum

**Affiliations:** 1 Department of Women’s and Children’s Health, Karolinska Institutet, Biomedicum A4, Tomtebodavägen 16, Solna, 17165 Stockholm, Sweden; 2 Laboratory of Tissue Engineering, Division of Pediatric Surgery, Department of Surgery and Transplantation, Copenhagen University Hospitalet Rigshospitalet, 2100 Copenhagen, Denmark; 3 Department of Clinical Medicine, Copenhagen University, 2200 Copenhagen, Denmark

**Keywords:** human, urinary bladder, microRNA, urothelium, bladder wound healing

## Abstract

Urinary bladder wound healing shares many features with skin healing, involving several molecular players, including microRNAs (miRs). This study investigated the role of miR-132 in urothelial cells. We analyzed miR-132 expression in rat bladder using in situ hybridization and conducted gain and loss of miR-132 function assays in primary human urothelial cells (HUCs). These assays included cell proliferation and migration studies. To explore the regulation of miR-132 expression, cells were treated with wound-healing-related factors such as interleukin 6 (IL-6), interleukin 10 (IL-10), and transforming growth factor beta-1 (TGF-β1). Predictive bioinformatics and a literature review identified potential miR-132 targets, which were validated through real-time polymerase chain reaction (RT-PCR) and Western blot analysis. miR-132 was found to promote cellular proliferation and migration during the early stages of urothelial wound repair. Its expression was modulated by key cytokines such as IL-6, IL-10, and TGF-β1. miR-132 played a crucial role in urothelial wound healing by enhancing cell proliferation and migration, regulated by cytokines, suggesting its action within a complex regulatory network. These findings highlight the therapeutic potential of targeting miR-132 in bladder injury repair, offering new insights into bladder repair mechanisms.

## 1. Introduction

MicroRNAs (miRs) are versatile non-coding RNA molecules implicated in gene regulation. They are short, about 22 nucleotides long, single-stranded molecules that mainly silence gene expression by repressing translation or targeting mRNA degradation [1]. According to the MirBase v.23 database (https://www.mirbase.org/), the human genome encodes approximately 2300 mature miRs [2,3], and it has been estimated that nearly all mRNA transcripts contain miR response elements encoded in their sequences [4]. MiRs control different biological processes, from cellular proliferation to differentiation and development; in addition, miRs can be involved in the progression and onset of pathological conditions such as cancer [5,6].

The repair process after injury of the urinary bladder involves various cell types as it leads to an inflammatory response, cell proliferation, local migration, the formation of granulation tissue, a new extracellular matrix, angiogenesis, apoptosis, and tissue remodeling that ends in scar formation [7,8]. The above-mentioned events are triggered upon injury and are controlled by a myriad of cellular signals involving cytokines and growth factors acting in a temporal manner. The fine-tuning of specific gene expressions is ultimately responsible for the progression of the process. MiRs are reported as key epigenetic factors that control healing by targeting genes involved in several cellular features such as epithelial and fibroblast cell proliferation and migration, angiogenesis, extracellular matrix production, apoptosis, and inflammation [9]. In the context of tissue injury, miRs have been proven to play a critical regulatory role in wound healing, particularly in the skin. Several studies have identified specific miRs that are involved in modulating the different processes of tissue repair. Given these insights, we previously aimed to investigate whether similar miRs were involved in the healing process of the urinary bladder, which shares some biological pathways with skin tissue repair. Our initial analysis identified a set of significantly expressed genes upon bladder wounding [10]. In addition, we identified a set of miRs that did not pass our stringent cut-off criteria but presented differential expression profiles that allowed us to clearly separate wounded from non-wounded animals; miR-132 was detected in the latter set of genes. 

MiR-132 was initially described as a miR related to neuronal function [11] and has been proposed as a potential therapeutical drug for preventing or treating neurogenerative disorders [12]. MiR-132 has also attracted attention for its role in wound healing. In cutaneous wounds, miR-132 plays an important role in transitioning from the inflammatory to the proliferative phase of healing by inhibiting inflammatory stimulus and increasing keratinocyte proliferation [13,14]. We hypothesized that miR-132 could be equally important in bladder wound healing. In this study, we therefore aimed at exploring the role of miR-132 in rat bladders in vivo and in human urothelial cells (HUCs) in vitro.

## 2. Results

### 2.1. MiR-132 Was Differentially Expressed during the Inflammatory and Proliferative Phases of the In Vivo Rat Wound Healing Process

**Figure 1:**
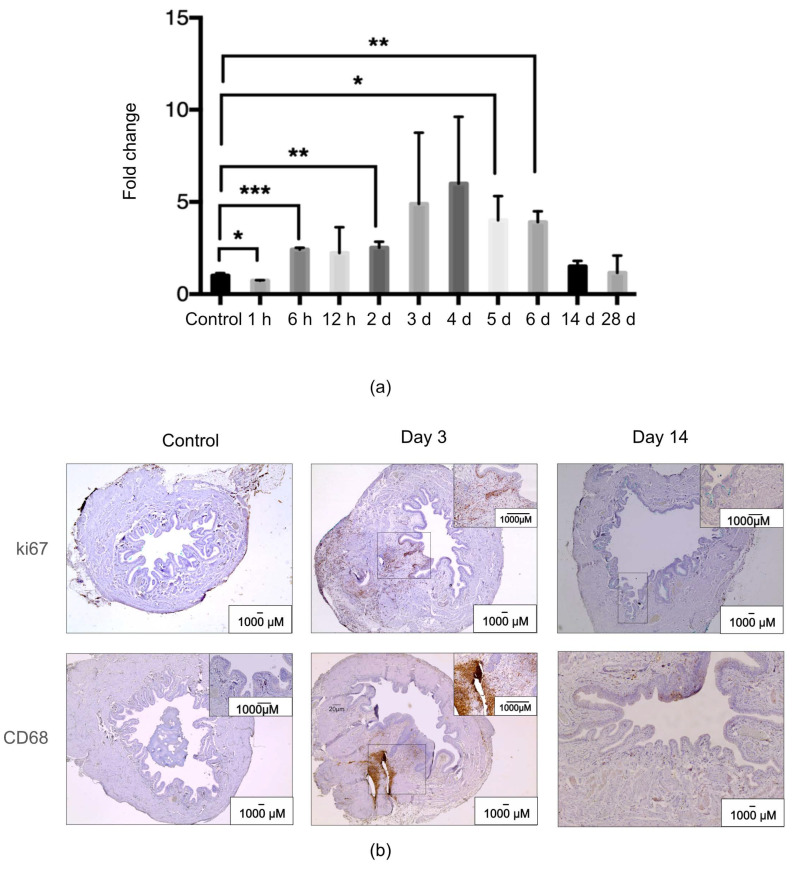
Expression of miR-132 during in vivo rodent bladder wound healing. (**a**) RNA from rodent urinary bladder samples (n = 4 rats, each time point) was analyzed using real-time polymerase chain reaction (RT-PCR) for miR-132 expression. (**b**) Representative histological sections showing proliferation (ki67) and inflammation (CD68) markers during the first and second weeks of healing. The values are expressed as a fold change relative to non-wounded control bladders. * *p* < 0.05, ** *p* < 0.01, *** *p* < 0.001.

First, we analyzed the expression pattern of miR-132 during urinary bladder healing using total RNA isolated from our previously published experiments [8,10]. Real-time polymerase chain reaction (RT-PCR) analysis indicated significant differences in the expression of this miR during the first week of wound healing (Figure 1a). In accordance with the RT-PCR, the miR-132 peak expression coincided with the inflammatory/proliferative phase of healing, as indicated by the immunohistochemical detection of both proliferative (ki67) and inflammatory markers (cd68) (Figure 1b). 

### 2.2. MiR-132 Was Mainly Upregulated in the Rat Bladder Urothelial Layer 

**Figure 2:**
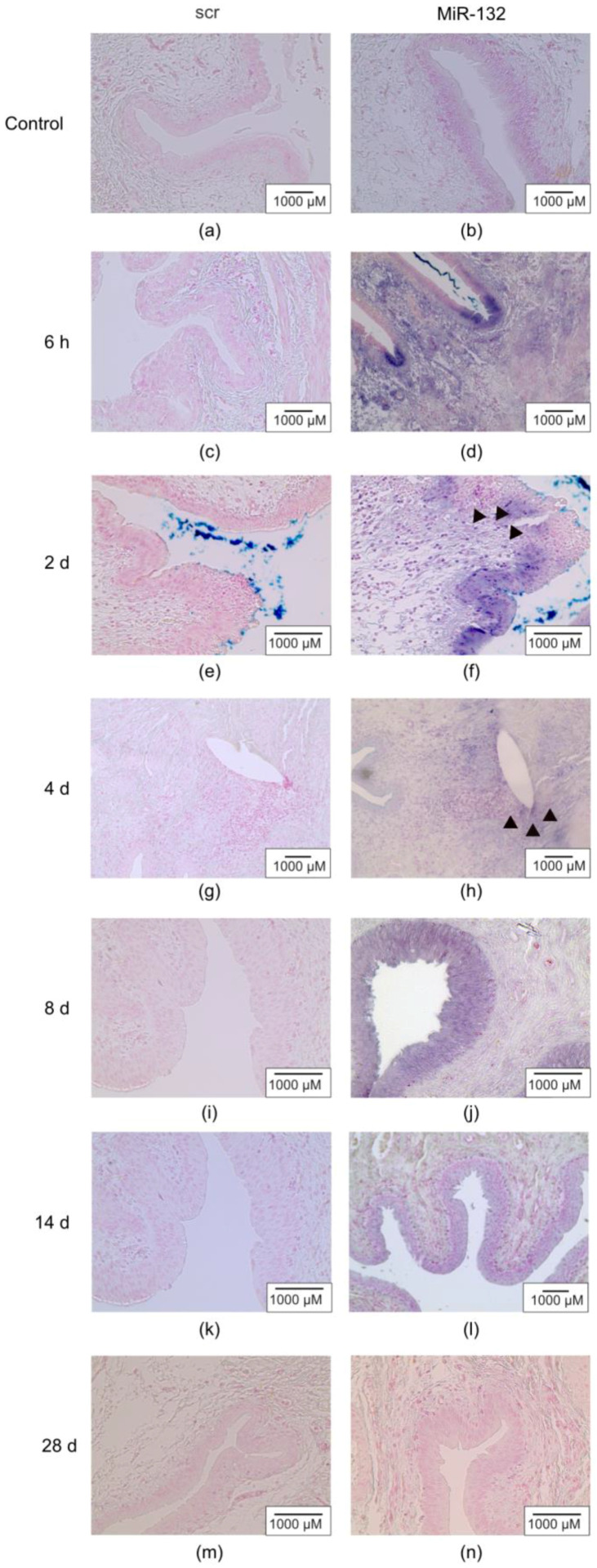
MiR-132 expression was mainly found in the mucosa during the first week of healing. In situ hybridization in non-wounded (control) and wounded bladders at (**a**,**b**) 6 h and (**e**–**n**) 2–28 days post wounding. (**a**,**c**,**e**,**g**,**i**,**k**,**m**) Digoxigenin (DIG)-labeled miRCURY LNA scramble probes (scr) or (**b**,**d**,**f**,**h**,**j**,**l**,**n**) probes specifically designed for miR-132 were used. MiR-132 (blue-purple color) was detected mainly in the urothelial cell layers, with some positive cells in the submucosa and around the suture areas, where persistent inflammation reaction was observed (dark triangles). Two out of four animals per time point and condition were analyzed.

In situ hybridization showed an early expression pattern of miR-132 (Figure 2a–n) located mainly in the epithelial portion of the bladder wall (Figure 2f), and some miR-132 expression was also detected in the submucosa and near suture areas with inflammatory cell infiltrate (Figure 2h).

### 2.3. MiR-132 Was Temporarily Upregulated upon In Vitro Scratch Wounding in HUCs

**Figure 3:**
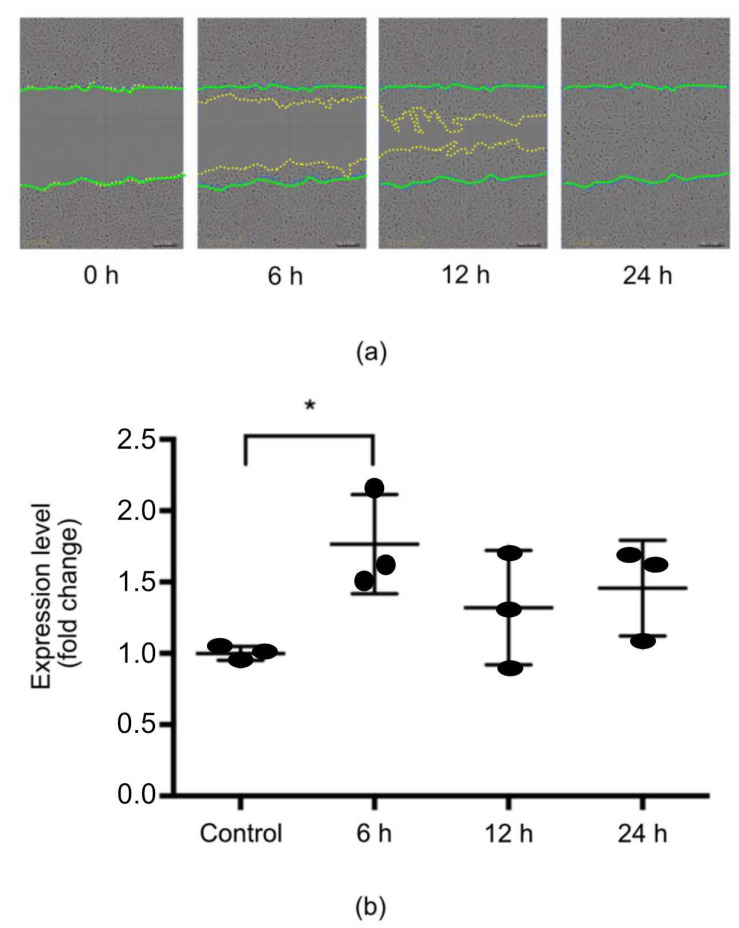
Human urothelial cells (HUCs) upregulated miR-132 expression after in vitro scratch wounding. (**a**) Representative HUC pictures illustrating the progression of an in vitro scratch wound. Green dotted lines represent original wound. Yellow dotted lines represent the area where the cells had migrated. (**b**) RT-PCR analysis of the expression of miR-132 in HUC upon wounding (n = 3). * *p* < 0.05.

Upon in vitro scratch wounding, primary urothelial cells significantly upregulated the expression of miR-132 at 6 h after wounding. The levels of miR-132 remained relatively higher than the controls at 12 and 24 h post wounding, but the differences at these later time points were not statistically significant (Figure 3a). At 24 h, the wound was completely closed (Figure 3b). These results indicated that miR-132 could be involved in an early epithelial response to wounding.

### 2.4. Expression of miR-132 in HUCs Was Regulated by Wound Healing Environment 

**Figure 4:**
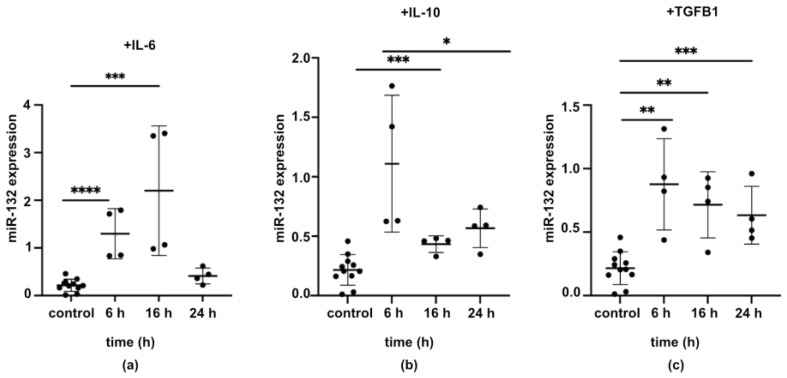
Wound healing-related signaling molecules that modulated miR-132 expression. Primary HUCs were treated with (**a**) interleukin 6 (IL-6), (**b**) interleukin 10 (IL-10), and (**c**) transforming growth factor beta-1 (TGF-β1) (n = 4). The expression of miR-132 was evaluated using digital PCR. * *p* < 0.05; ** *p* < 0.01; *** *p* < 0.001; **** *p* < 0.0001.

Next, to detect the possible factors contributing to the differential expression of miR-132 in epithelial healing, we treated urothelial cells with three known factors that are associated with wound healing, i.e., interleukin 6 (IL-6), interleukin 10 (IL-10), and transforming growth factor beta-1 (TGF-β1) (Figure 4a–c), and analyzed miR-132 expression at 6, 16, and 24 h. We found that miR-132 expression was upregulated by all these cytokines in a time-dependent manner. IL-6 induced an increase in miR-132 levels at 6 and 16 h, returning to almost basal levels after 24 h (Figure 4a). Elevated levels of miR-132 were detected even after 24 h of stimulation with IL-10 (Figure 4b) and TGF-β1 (Figure 4c).

### 2.5. MiR-132 Affected the Proliferation of Human Bladder Epithelial Cells 

**Figure 5:**
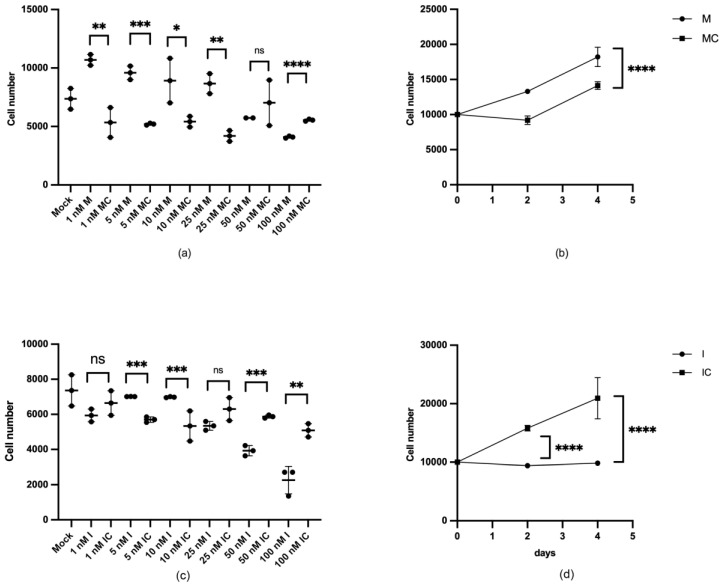
Gain in miR-132 expression induced an increase in cell proliferation. Primary HUCs transfected with transfection reagent alone (mock), with different specific RNA molecules designed to (**a**,**b**) mimic or (**c**,**d**) inhibit endogenous miR-132 expression and their respective controls (**c**,**d**). A total of 10000 cells/well were transfected with 5 nM mimic or 50 nM inhibitor and their respective controls. The total number of cells were estimated after 2 and 4 days in culture (ns = not significant; * *p* < 0.05; ** *p* < 0.01; *** *p* < 0.001; **** *p* < 0.0001, n = 3).

To determine the biological process in which miR-132 may be involved, we investigated its role in cellular proliferation and migration. The gain and loss of expression of miR-132 with specific miR-132 double-stranded RNA mimics and inhibitors indicated that miR-132 was involved in HUC proliferation (Figure 5). Transfection of HUC with 1–25 nM miR-132 mimics induced a significant increase in cell proliferation (Figure 5a), compared to cells transfected with transfection reagent alone or with control oligonucleotides (negative controls). Higher concentrations of mimic molecules (50 and 100 nM) did not enhance proliferation but rather seemed to have the opposite effect. In contrast, transfection with miR-132 specific inhibitors did not have an obvious effect on cell proliferation when used at low concentrations (1–25 nM), but transfection with 50 and 100 nM of an miR-132 inhibitor significantly reduced the proliferative capacity (Figure 5c). When evaluating the long-term effect (on days 2 and 4) on the proliferation of cells transfected with 5 nM mimic or with 50 nM inhibitor, a sustained positive effect was observed on proliferation with mimic transfection (Figure 5b), whereas cells transfected with 50 nM inhibitor exhibited low proliferation during the whole analysis period (Figure 5d).

### 2.6. Overexpression of MiR-132 Increased In Vitro HUC Migration

**Figure 6:**
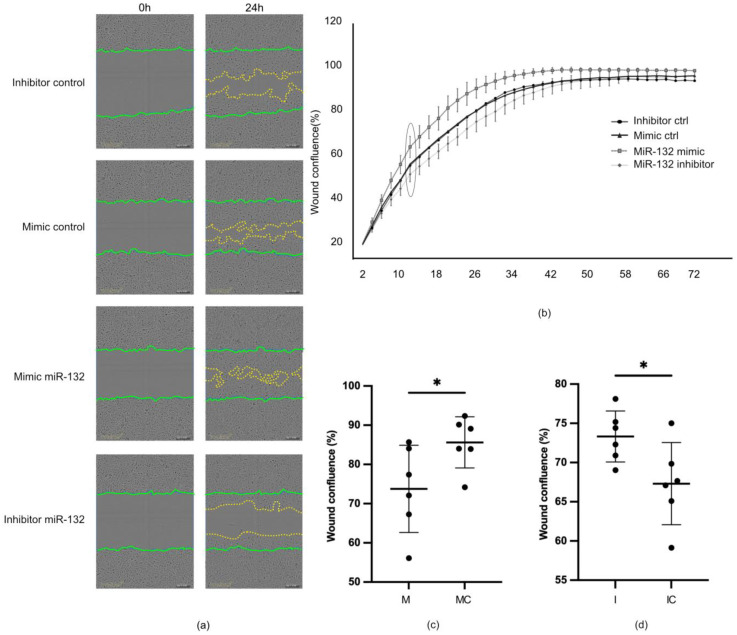
Gain in miR-132 expression accelerated in vitro wound healing. Gain and loss of function experiments using mimic or inhibitor molecules for miR-132 and respective controls. (**a**) Representative pictures showing the scratch area at time 0 and 24 h post wounding. Green lines marking the initial wound edges and yellow lines marking the wound edges 24 h later (**b**) Graph representing the average percentage of wound confluency and standard deviations over time in each experimental condition, performed in 6 replicates each. (**c**,**d**) Graph showing the percentage of wound confluence after different treatments 12 h after wounding (ns = not significant; * *p* < 0.05. Representative data from one of a total three experiments are presented.

To explore whether the overexpression of miR-132 affects the cells’ migratory capacity, we next performed scratch wound healing assays on HUCs transfected with miR-132 mimics and inhibitors and their corresponding controls. Cells transfected with miR-132 mimics closed the wound faster than those transfected with the corresponding negative control (*p* < 0.01). Furthermore, transfection of the cells with miR-132 inhibitory molecules delayed the wound closure (Figure 6a–d).

### 2.7. MiR-132 Regulated RAS p21 Protein Activator 1 (RASA1), Phosphatase and Tensin Homolog (PTEN), and Mitogen-Activated Protein Kinase (MAPK) in HUCs

To further investigate the possible mechanisms responsible for the miR-132-mediated effects in cell proliferation and migration, we combined computational prediction and experimental validation of miR-132 target genes using the miRTarBase [15]. MiRTarBase exploration showed 246 potential genes with validated miR-132–target interactions. To narrow down the list of miR-132 target genes that could be mediating the observed effects in cell proliferation/migration, we investigated which of the putative genes had been previously associated with wound healing. MiR-132 had been shown to be a positive regulator of skin wound healing [16] and to promote proliferation and tube formation in endothelial cells [17] by targeting RASA1. MiR-132 had also been shown to modulate pancreatic beta cell proliferation via MAPK and PTEN downregulation [18]. We therefore investigated the effect of miR-132 on the mRNA expression of RASA1, PTEN, and MAPK and the corresponding protein levels in HUC. 

**Figure 7:**
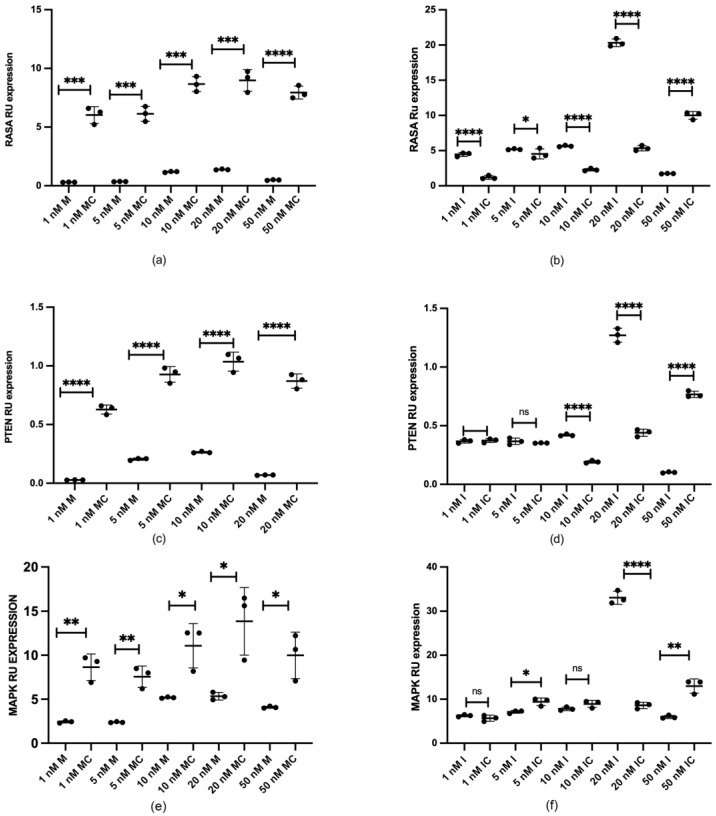
MiR-132 modulated the mRNA expression of RAS p21 protein activator 1 (RASA1), Phosphatase and tensin homolog (PTEN), and Mitogen-activated protein kinase (MAPK). Quantitative RT-PCR analysis of the mRNA expression of HUCs upon transfection with the indicated concentrations of (**a**,**c**,**e**) mimic “M” and its corresponding control “MC” and (**b**,**d**,**f**) inhibitor “I” and its corresponding control “IC”. Graphs show average gene expression. (ns = not significant; * *p* < 0.05; ** *p* < 0.01; *** *p* < 0.001; **** *p* < 0.0001).

To investigate the potential regulation of RASA1, PTEN, and MAPK mRNAs by miR-132 in HUCs, we performed gain and loss of function experiments. Overexpression of miR-132 was achieved by transfecting HUCs with miR-132 mimics at final concentrations of 1, 5, 10, 20, and 50 nM, resulting in the decreased expression of these mRNAs compared to control transfected cells (Figure 7a,c,d). Conversely, miR-132 inhibitors at the same concentration induced an increase in the expression levels of the above-mentioned mRNAs (Figure 7b,d,f), except for the highest concentration of miR132 inhibitors (50 nM), where the opposite results were observed.

**Figure 8:**
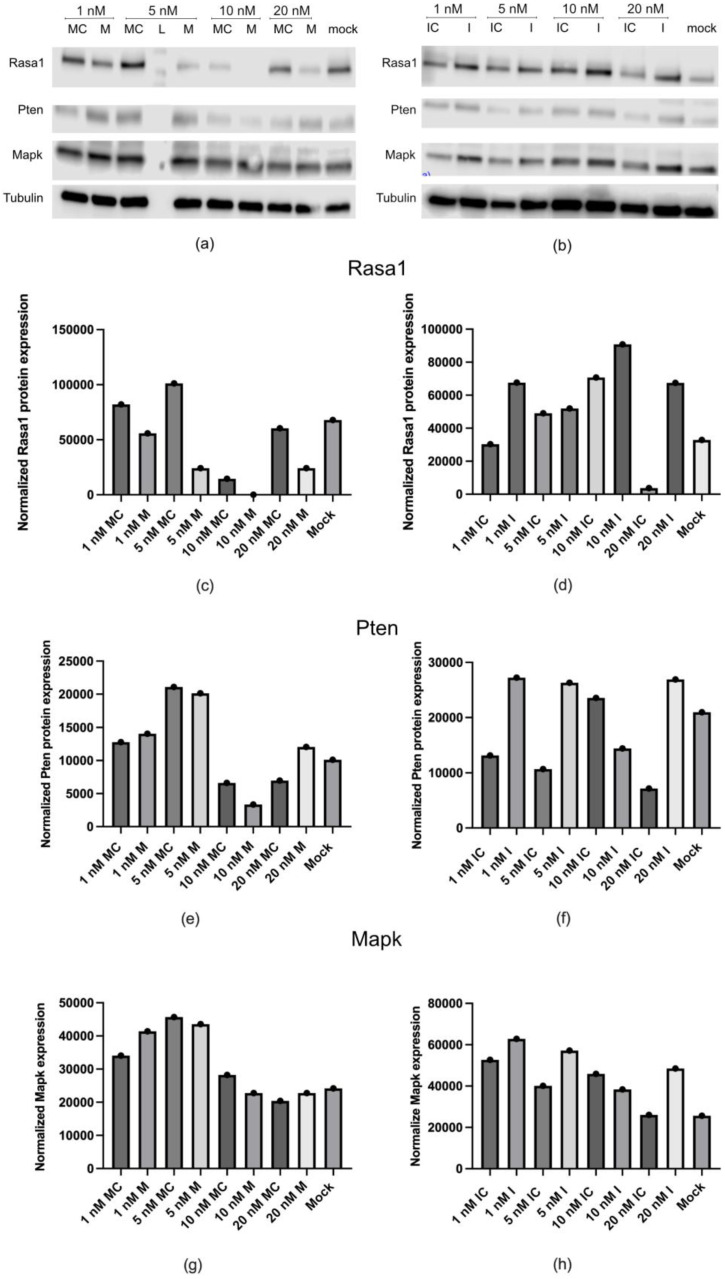
Analysis of *RASA1*, *PTEN*, and *MAPK* protein expression in human primary urothelial cells. Representative image showing one of a total of two performed experiments analyzing protein expression with Western blot after transfection with transfection reagent alone (mock) or with specific double- and single-stranded RNA molecules designed to mimic “M” (**a**,**c**,**e**,**g**) or inhibit “I” (**b**,**d**,**f**,**h**) endogenous miR-132 and corresponding controls for mimic “MC” and inhibitor. L denotes protein padder sample. Lower bars represent normalized values to the housekeeping protein tubulin (one of the two experiments is represented).

Next, we evaluated whether the observed decrease in mRNA expression was reflected at the protein level (Figure 8 and Supplementary Figure S1). We found that the inhibition of miR-132 increased the endogenous protein levels of *RASA1* at all tested concentrations (Figure 8d), correlating well with the mRNA data. However, the same did not occur for either *PTEN* or *MAPK* (Figure 8f,h), with an irregular pattern of protein expression. Furthermore, although the overexpression of miR-132 decreased the *RASA1* protein levels (Figure 8c and Supplementary Figure S1), it did not have a clear effect on *MAPK* and *PTEN* protein expression (Figure 8e,g). 

Together, these results indicated that miR-132 regulates both RASA1 mRNA and protein degradation, while it affects PTEN and MAPK mainly by mRNA degradation or decreased translation.

## 3. Discussion

In this study, we investigated the role of miR-132 in urinary bladder wound healing. We found that there was a significant upregulation of this miR during the first week of rodent bladder wound healing, suggesting its potential involvement in the early stages of tissue repair. Through in situ hybridization, we found that the cells within the rat bladder that mainly expressed miR-132 were urothelial cells. To further elucidate its translational and functional role, we conducted in vitro assays on primary HUCs, focusing on urothelial cell wound responses: proliferation and migration. We found that miR-132 was temporally upregulated after in vitro scratch wounding. Furthermore, the stimulus of a wound-enriched environment for the urothelial cells, such as exposure to cytokines IL-6, IL-10, and TGF-β1, induced expression of miR-132 in these cells. MiR-132 gain and loss of function experiments, using specific mimic and inhibitor oligonucleotides, showed that miR-132 induced both urothelial cell proliferation and migration in vitro. Furthermore, by using bioinformatic target predicting tools and a literature review, we identified three possible targets for miR-132 in urothelial cells. All together, these assays demonstrated that miR-132 influenced primary urothelial cell proliferation and migration, indicating its importance in the process of healing.

MiR-132 is a versatile miR with significant roles in various biological processes, from neural function [15] to immune responses and tissue repair [16,17,18]. Our findings were in agreement with studies reporting a pivotal role of this miR in skin wound healing, such as its key functions in modulating the transition from the inflammatory phase to the proliferative phase of wound healing [13], by regulation of a large number of immune response- and cell cycle-related genes, thereby reducing an excessive inflammatory response and stimulating proliferation. 

The early upregulation of miR-132 post injury indicated its role as an early response element. This early upregulation might be crucial for initiating the repair process. The upregulation of miR-132, induced by Il-6, IL-10, and TGF-β1, suggests that this miR acts within a complex network modulated by these cytokines.

To understand the molecular mechanisms by which miR-132 modulated the observed effects, we used miRTarBase for miR target prediction. This computational tool uses algorithms to predict miR binding sites on target mRNAs based on sequence complementarity and other factors such as evolutionary conservation. Furthermore, the database contains information on the level and type of experimental evidence for each of the predicted targets, facilitating the identification of strong candidate genes. 

In our study, we combined targeted miR prediction with literature reviews to identify putative targets of miR-132. This approach led us to focus on RASA1, PTEN, and MAPK as potential targets. While we validated the relationship between miR-132 and these genes through mRNA expression analyses, there were differences in the level of regulation of the three genes. 

RASA1 was regulated by miR-132 at both the mRNA and protein levels, while MAPK and PTEN did not demonstrate clear protein regulation by miR-132. Specifically, inhibition of miR-132 led to an upregulation of these genes at both the mRNA and protein levels, suggesting that miR-132 represses MAPK and PTEN under normal conditions. However, the introduction of a miR-132 mimic did not significantly downregulate *MAPK* or *PTEN*, which may be due to compensatory regulatory mechanisms or the stable nature of MAPK and PTEN proteins.

Taken together, this suggested that miR-132 regulation may mainly impact mRNA degradation rather than protein abundancy. Further investigations are needed to determine the exact regulatory mechanisms involved.

Furthermore, one study limitation is that our approach to select the possible miR-132 target genes was inherently selective, focusing on already-known or predicted interactions. To gain a more comprehensive understanding of miR-132’s target repertoire, a proteomic or whole-genome approach could allow for the unbiased identification of both direct and indirect targets.

Furthermore, we lacked functional assays directly linking the observed increase in cell proliferation and migration with the activity of these target genes. Although we demonstrated changes in the expression patterns of RASA1, PTEN, and MAPK, we did not establish a direct causal relationship between these changes and the enhanced proliferative and migratory behaviors. RASA1 has been shown to inhibit cell migration by competing with Rab21, a GTPase that binds to integrins and regulates receptor trafficking [19]. The other two target genes, PTEN [20] and MAPK [21], regulate the cell cycle. 

The limitations of this study highlight the need for further research to establish the functional significance of these targets in the context of miR-132-mediated bladder wound healing. Future studies should include assays that directly measure the impact of modulating these genes on cellular functions related to wound healing, such as proliferation and migration. Techniques such as gene knockdown or overexpression followed by detailed phenotypic analyses would provide more robust evidence of the roles these targets play in miR-132 function.

The role of miR-132 in bladder tissue has been previously studied in the context of bladder outlet obstruction, implying that this miR acts on tissue remodeling and fibrosis [22]. To our knowledge, the role of miR-132 in urothelial healing is new.

Given its role in cell proliferation and migration, miR-132 could be a promising therapeutic target in cases of impaired bladder wound healing, bladder inflammatory diseases, and fibrosis. Modulating its expression through miR mimics or inhibitors, particularly in the early stages of injury, could enhance the healing process. However, precise control would be necessary to avoid excessive cell proliferation or migration, which could lead to complications such as hypertrophic scarring or improper tissue regeneration.

**Figure 9:**
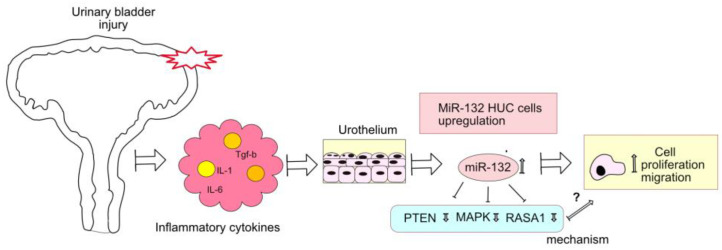
Simplified model of miR-132 in bladder injury repair. This figure illustrates the role of miR-132 in urothelial cells following urinary bladder injury. Upon injury, a cascade of molecular signals and cellular responses is triggered. An increase in inflammatory cytokines (pink), such as IL-6, IL-10, and TGF-β1, act upon the urothelium. This leads to the upregulation of miR-132. MiR-132 targets specific genes such as RASA1, PTEN, and MAPK (light-green box), which are crucial for cellular processes during repair. The downregulation of these target genes by miR-132 enhanced cell proliferation and migration (light-yellow box) and could thereby contribute to the repair mechanisms following bladder injury. The mechanism by which this genes mediate miR-132 effects need to be further explored (denoted by the question mark).

Our study provided valuable insights into the regulation and potential targets of miR-132 in bladder wound healing, and highlighted the need for comprehensive functional validations to fully elucidate the molecular mechanisms at play. Addressing these gaps would further enhance our understanding of miR-132’s and other miRs’ therapeutic potential and their role in tissue regeneration (Figure 9).

## 4. Materials and Methods

### 4.1. Ethics 

The in vivo urinary bladder wound healing study was approved by the national animal ethics laws and institutional regulations on animal studies (Stockholm County Committee on Animal Research, 20150703 Reg. N107/15) [8]. The animal interventions and housing complied with veterinary best practice norms and the ARRIVE guidelines at the Unit for Comparative Medicine, Karolinska Institutet, Solna, Sweden.

### 4.2. RNA Extraction

Rodent bladder RNA was isolated as previously described [8,10]. In brief, RNA was extracted using a TissueLyser (Qiagen, Hilden, Germany) for tissue homogenization and Qiazol Lysis Reagent® (Thermo Fisher Scientific, Waltham, MA, USA). After extraction, the concentration of total RNA was determined using a NanoDrop® ND-1000 Spectrophotometer (NanoDrop Technologies Inc., Wilmington, DE, USA).

### 4.3. RT-PCR Analysis 

For each sample, 20 ng of total RNA was reverse-transcribed into complementary DNA (cDNA) using the TaqMan microRNA Reverse Transcription Kit and the multiplex RT primer pool containing miR-specific stem-loop primers (Thermo Fisher Scientific, Foster City, CA, USA) as previously described [10]. The expression of miR-132 was determined using TaqMan expression assays (Thermo Fisher Scientific Foster City, CA, USA) and normalized based on the values of U6 small nuclear RNA (rat samples) or RNU48 (human samples). Detailed information for the TaqMan assays used in this study is provided in Table 1.

To quantify mRNAs, total RNA was reverse-transcribed using the RevertAid First Strand cDNA Synthesis Kit (Thermo Fisher Scientific, Foster City, CA, USA). RASA1, MAPK, and PTEN mRNAs were quantified using TaqMan gene expression assays. Target gene expression levels were normalized between samples to the internal control 18 S rRNA (forward: 5′-CGGCTACCACATCCAAGGAA-3′; reverse: 5′-GCTGGAATTACCGCGGCT-3′; probe: 5′-FAM-TGCTGGCACCAGACTTGCCCTC-TAMRA-3′).

### 4.4. In Situ Hybridization in Rat Urinary Bladders 

For in situ hybridization, we used miRCURY LNA™ miR detection probes (Qiagen, Hilden, Germany) and followed the manufacturer’s instructions and previously published [13]. In brief, formalin-fixed paraffin-embedded rat bladder sections (6 μm thickness) were deparaffinized and washed followed by 3 min treatment with proteinase K (Qiagen, Hilden, Germany) (2 μg/mL) at 37 °C. After 30 min in the pre-hybridization buffer, the sections were hybridized with a miR-132-specific or scramble digoxigenin (DIG)-labeled miRCURY locked nucleic acid probe (25 nM) (Exiqon Woburn, MA, USA) for 2 h at 50 °C. Then, the slides were washed under stringent conditions with saline–sodium citrate buffer (SSC) pre-warmed at 55 °C. The sections were then incubated with alkaline phosphatase (AP)-conjugated anti-DIG antibody (1:1000) (Roche, Mannheim, Germany) for 1.5 h at room temperature. The probe was visualized by adding freshly prepared AP substrate (Roche, Mannheim, Germany) to the sections and incubated at room temperature for 2–12 h in a humidity chamber. Thereafter, the sections were washed and mounted.

### 4.5. Histological Analysis

Immunostainings were performed with 5 μm paraffin-embedded sections as previously described [8]. Briefly, after deparaffinization and tissue hydration, antigen retrieval was performed with citrate buffer at 95 °C for 20 min. Endogenous peroxidase activity was quenched by immersing in 3% H_2_O_2_ (Sigma, St. Louis, MO, USA)/methanol for 10 min. Non-specific binding was blocked by incubation with 4% of normal serum (Vector Laboratories Burlingame, CA, USA) in tris-buffered saline (TBS) for 30 min at room temperature. Immunodetection was performed with the following antibodies at 4 °C overnight: CD68 (inflammation marker, cat.ab31630, Abcam, Cambridge, UK) and Ki67 (proliferative cells, cat.ab16667, Abcam, Cambridge, UK), followed by incubation with rabbit- or rat-absorbed horseradish peroxidase-conjugated (HRP)-conjugated goat anti-mouse IgG secondary antibody (Vector Laboratories), respectively, for 1 h at room temperature. Antibody binding was viewed after the incubation of slides with 3-amino-9-ethylcarbazole or 3,3′-diaminobenzidine HRP substrate (Vector Laboratories) for 5 min. The slides were then counterstained with hematoxylin, dehydrated in graded alcohol, cleared in X-TRA-Solv (J.T. Baker, Burgdorf, Germany) and mounted with mounting medium, X-TRA-Kit (J.T. Baker, Burgdorf, Germany).

### 4.6. HUC Culture

Human bladder urothelial cells were purchased from Science Research Laboratories. Cells were cultured on poly-L-lysine-coated culture flasks at the recommended minimal density (5000 cells/cm^2^) in urothelial cell medium (UCM, catalog #4321) and growth factor supplements (catalog #4352) as previously published [10]. Cells were used for a maximum of four passages.

### 4.7. In Vitro 2D Wound Healing Assay in HUCs

Primary HUCs were seeded into poly-L-lysine-coated culture dishes at a density of 40,000 cells/cm^2^. Once the cells formed a 100% confluency, the monolayers were wounded using a p200 pipet tip. Seven longitudinal and seven vertical straight lines were created. After washing once with PBS, 2 mL of UCM was added to the plates. RNA was extracted after 6, 12, and 24 h for further RNA expression analysis.

### 4.8. Urothelial Cell Treatments 

Before stimulus, HUCs were placed for 12 h in UCM without supplements. HUCs were then stimulated with cytokines and growth factors (all from ImmunoTools, Friesoythe, Germany) at the following concentrations: IL-10 (20 ng/mL), IL-6 (10 ng/mL), and TGF-β1 (10 ng/mL).

### 4.9. Cell Transfection 

Cells were transfected with MirVana for Hsa-miR-132 mimic (hsa-miR-132-3p IDMC10166, inhibitor (hsa-miR-132-3p ID:MH10166), and their controls (Thermo Fisher Scientific, *mir*Vana™, Ambion™) by using Lipofectamine™ RNAiMAX Transfection Reagent and following the specifications of the manufacturer (Thermo Fisher Scientific, Foster City, CA, USA). The mimic double-stranded RNA molecules are designed to mimic endogenous MiR-132, resulting in downregulation of target mRNA targets. Inhibitors are single-stranded oligonucleotides designed to bind and inhibit endogenous miR-132. As the negative control, we used mirVana miRNA Mimic Negative Control #1 (Cat n# 4464058 Thermo Fisher Scientific, Foster City, CA, USA) and *mir*Vana miRNA Inhibitor Negative Control #1 (Cat # 4464076 Thermo Fisher Scientific, Foster City, CA, USA).

### 4.10. MiR-132 Gene Target Selection

To find genes targeted by miR-132 related to wound healing and bladder tissue repair, we explored the public database for prediction that is available in the miRTarBase (https://doi.org/10.1093/nar/gkab1079). MiRTarBase revealed experimentally validated target genes for human miR-132. Three genes were selected based on a high score of interaction and because they had been previously studied in scientific articles [23,24].

### 4.11. Analysis of Cell Migration

After transfections with mimics and inhibitors for miR-132, HUCs were plated in IncuCyte^®^ ImageLock 96-well plates (Essen Bioscience, Ann Arborm MI, USA, catalog number: 4379) at a total density of 40000 cells/well as previously described [25]. Twenty-four hours later, the IncuCyte^®^ WoundMaker was used to create a standardized homogeneous wound in each well. Each plate was then placed inside the IncuCyte^®^ live-cell analysis system (Essen Bioscience; model: IncuCyte^®^ ZOOM), and the wounds were monitored every two hours. Initial scratch wound area, scratch wound area, and confluence of the wounded area were obtained. The images were analyzed with the IncuCyte^®^ Cell Migration Analysis software (nr. 9600-0012, Incucyte Zoom 2018A GUI) as previously described [26]. The data were presented as the percentage of wound confluence over time. Comparisons of migration rates were performed at 6–24 h post wounding. Representative images at each analyzed time point are included in Figure 6.

### 4.12. Metabolic Proliferation Assays

The effect of the gain or loss of miR-132 expression in HUC proliferation was evaluated using the Cell Counting Kit-8 (Sigma), by following the manufacturer’s instructions. In brief, 24 h after transfection, the cells were harvested and counted using Countess® Cell Counting Chamber slides (Thermo Fisher Scientific, Foster City, CA, USA). The standard curve was generated with a known amount of HUCs that were plated at the beginning of the experiment. Five thousand cells/well were plated into each well using 6 replicates per condition. The absorbance at 450 nm was measured in all the wells including transfected cells. The total number of cells was calculated in relation to the generated standard curve. 

### 4.13. Western Blot

HUCs were transfected with MirVana for Hsa-miR-132 mimic, inhibitor, and their corresponding controls (Thermo Fisher Scientific, *mir*Vana™, Ambion™), as mentioned above. Forty-eight hours after transfection, the total protein was collected using radioimmunoprecipitation assay buffer consisting of 150 mM NaCl, 1.0% IGEPAL^®^ CA-630, 0.5% sodium deoxycholate, 0.1% SDS, and 50 mM tris, pH 8 (Sigma Aldrich, Darmstadt, Germany) and in the presence of proteinase (Complete™ ULTRA Tablets, Mini, EASYpack Protease Inhibitor Cocktail, Roche, Basel, Switzerland) and phosphatase inhibitor cocktails (PhosStop Roche, Basel, Switzerland). Total protein concentration was evaluated using the bicinchoninic acid assay as per the manufacturer’s instructions (Thermo Fisher Scientific, Waltham, MA, USA). Then, 20 µg of total protein was loaded into 7.5% SDS-polyacrylamide electrophoresis gel (Mini-PROTEAN^®^ TGX™ Precast Gels, Bio-Rad, Hercules, CA, USA) and transferred onto polyvinylidene fluoride membranes (Trans-Blot Turbo Transfer System, Bio-Rad, Hercules, CA, USA) as previously described [8]. The membranes were thereafter blocked with TBS containing 5% bovine serum albumin and incubated overnight with a 1:1000 dilution of primary antibodies specific to β-tubulin, RASA1, PTEN, and MAPK, followed by 2 h of HRP secondary antibody incubation.

### 4.14. Statistical Analysis

Each experiment was performed with a minimum of 3 different biological replicates and 3 technical replicates. For the in vivo rat wound healing samples, a non-parametric Mann–Whitney *U* test was used to calculate the differences in gene expression. By comparing normalized gene expression values from each biological replicate, *p*-values < 0.05 were considered significant. Experiments involving in vitro cultured cells, *t*-test, and ANOVA tests were performed using GraphPad Prism version 10.3.1 for macOS (GraphPad Software, Boston, MA, USA, www.graphpad.com).

## 5. Conclusions

In summary, our findings demonstrated that miR-132 was an important mediator in urothelial wound healing driven by cytokine regulation. Targeting miR132 could offer novel therapeutic strategies for enhancing bladder repair and treating injuries. Further studies should aim at elucidating the exact mechanisms behind the observed miR-132 effects in cellular proliferation and migration, and explore the in vivo implications of modulating this miR.

## Figures and Tables

**Table 1:** Details of Taqman assays.

TaqMan Assay	Mature miR	Assay ID
U6 snRNA	GTGCTCGCTTCGGCAGCACATATACTAAAATTGGAACGATACAGAGAAGATTAGCATGGCCCCTGCGCAAGGATGACACGCAAATTCGTGAAGCGTTCCATATTTT	001973
RNU48	GATGACCCCAGGTAACTCTGAGTGTGTCGCTGATGCCATCACCGCAGCGCTCTGACC	001006
hsa-miR-132	UAACAGUCUACAGCCAUGGUCG	00457
RAS p21 protein activator 1 (RASA1)	-	Hs00243115
Phosphatase and tensin homolog (PTEN)	-	Hs02621230
Mitogen-activated protein kinase (MAPK)	-	Hs01046830

## Data Availability

A database with differentially expressed miR can be found at Gene Expression Omnibus (GSE176515). All the raw data generated in this publication are available upon request to the authors.

## Supplementary Materials

The following supporting information can be downloaded at: https://www.mdpi.com/article/10.3390/ijms252011039/s1.

## References

[1] Jonas S., Izaurralde E. (2015). Towards a molecular understanding of microRNA-mediated gene silencing. Nat. Rev. Genet..

[2] Plotnikova O., Baranova A., Skoblov M. (2019). Comprehensive Analysis of Human microRNA-mRNA Interactome. Front. Genet..

[3] Kozomara A., Griffiths-Jones S. (2011). miRBase: Integrating microRNA annotation and deep-sequencing data. Nucleic Acids Res..

[4] Friedman R.C., Farh K.K., Burge C.B., Bartel D.P. (2009). Most mammalian mRNAs are conserved targets of microRNAs. Genome Res..

[5] Ali Syeda Z., Langden S.S.S., Munkhzul C., Lee M., Song S.J. (2020). Regulatory Mechanism of MicroRNA Expression in Cancer. Int. J. Mol. Sci..

[6] Smolarz B., Durczynski A., Romanowicz H., Szyllo K., Hogendorf P. (2022). miRNAs in Cancer (Review of Literature). Int. J. Mol. Sci..

[7] Eming S.A., Martin P., Tomic-Canic M. (2014). Wound repair and regeneration: Mechanisms, signaling, and translation. Sci. Transl. Med..

[8] Chamorro C.I., Reinfeldt Engberg G., Fossum M. (2020). Molecular and histological studies of bladder wound healing in a rodent model. Wound Repair. Regen..

[9] Bushati N., Cohen S.M. (2007). microRNA functions. Annu. Rev. Cell Dev. Biol..

[10] Chamorro C.I., Eisfeldt J., Willacy O., Juul N., Fossum M. (2021). A database on differentially expressed microRNAs during rodent bladder healing. Sci. Rep..

[11] Rafat M., Moraghebi M., Afsa M., Malekzadeh K. (2021). The outstanding role of miR-132-3p in carcinogenesis of solid tumors. Hum. Cell.

[12] El Fatimy R., Li S., Chen Z., Mushannen T., Gongala S., Wei Z., Balu D.T., Rabinovsky R., Cantlon A., Elkhal A. (2018). MicroRNA-132 provides neuroprotection for tauopathies via multiple signaling pathways. Acta Neuropathol..

[13] Li D., Wang A., Liu X., Meisgen F., Grunler J., Botusan I.R., Narayanan S., Erikci E., Li X., Blomqvist L. (2015). MicroRNA-132 enhances transition from inflammation to proliferation during wound healing. J. Clin. Investig..

[14] Li X., Li D., Wang A., Chu T., Lohcharoenkal W., Zheng X., Grunler J., Narayanan S., Eliasson S., Herter E.K. (2017). MicroRNA-132 with Therapeutic Potential in Chronic Wounds. J. Investig. Dermatol..

[15] Qian Y., Song J., Ouyang Y., Han Q., Chen W., Zhao X., Xie Y., Chen Y., Yuan W., Fan C. (2017). Advances in Roles of miR-132 in the Nervous System. Front. Pharmacol..

[16] Cui J., Zheng W., Sun Y., Xu T. (2022). Inducible MicroRNA-132 Inhibits the Production of Inflammatory Cytokines by Targeting TRAF6, TAK1, and TAB1 in Teleost Fish. Infect. Immun..

[17] Ge L., Wang K., Lin H., Tao E., Xia W., Wang F., Mao C., Feng Y. (2023). Engineered exosomes derived from miR-132-overexpresssing adipose stem cells promoted diabetic wound healing and skin reconstruction. Front. Bioeng. Biotechnol..

[18] Ma T., Chen Y., Chen Y., Meng Q., Sun J., Shao L., Yu Y., Huang H., Hu Y., Yang Z. (2018). MicroRNA-132, Delivered by Mesenchymal Stem Cell-Derived Exosomes, Promote Angiogenesis in Myocardial Infarction. Stem Cells Int..

[19] Mai A., Veltel S., Pellinen T., Padzik A., Coffey E., Marjomaki V., Ivaska J. (2011). Competitive binding of Rab21 and p120RasGAP to integrins regulates receptor traffic and migration. J. Cell Biol..

[20] Brandmaier A., Hou S.Q., Shen W.H. (2017). Cell Cycle Control by PTEN. J. Mol. Biol..

[21] Cargnello M., Roux P.P. (2011). Activation and function of the MAPKs and their substrates, the MAPK-activated protein kinases. Microbiol. Mol. Biol. Rev..

[22] Sadegh M.K., Ekman M., Krawczyk K., Svensson D., Goransson O., Dahan D., Nilsson B.O., Albinsson S., Uvelius B., Sward K. (2015). Detrusor induction of miR-132/212 following bladder outlet obstruction: Association with MeCP2 repression and cell viability. PLoS ONE.

[23] Li X., Li D., Wikstrom J.D., Pivarcsi A., Sonkoly E., Stahle M., Landen N.X. (2017). MicroRNA-132 promotes fibroblast migration via regulating RAS p21 protein activator 1 in skin wound healing. Sci. Rep..

[24] Mziaut H., Henniger G., Ganss K., Hempel S., Wolk S., McChord J., Chowdhury K., Ravassard P., Knoch K.P., Krautz C. (2020). MiR-132 controls pancreatic beta cell proliferation and survival through Pten/Akt/Foxo3 signaling. Mol. Metab..

[25] Juul N., Willacy O., Mamand D.R., El Andaloussi S., Eisfeldt J., Chamorro C.I., Fossum M. (2023). Insights into cellular behavior and micromolecular communication in urothelial micrografts. Sci Rep..

[26] Sun M., Rethi B., Krishnamurthy A., Joshua V., Wahamaa H., Catrina S.B., Catrina A. (2021). An Image-based Dynamic High-throughput Analysis of Adherent Cell Migration. Bio-Protocol.

