# Supplementary material for: Exploring the Role of miR-132 in Rat Bladders and Human Urothelial Cells during Wound Healing
Source: Int J Mol Sci. 2024 Oct 14;25(20):11039. doi: 10.3390/ijms252011039 (PMC11508042; doi:10.3390/ijms252011039)
Supplement: Supplementary file 1 [file ijms-25-11039-s001.zip › ijms-3121613-supplementary.pdf]

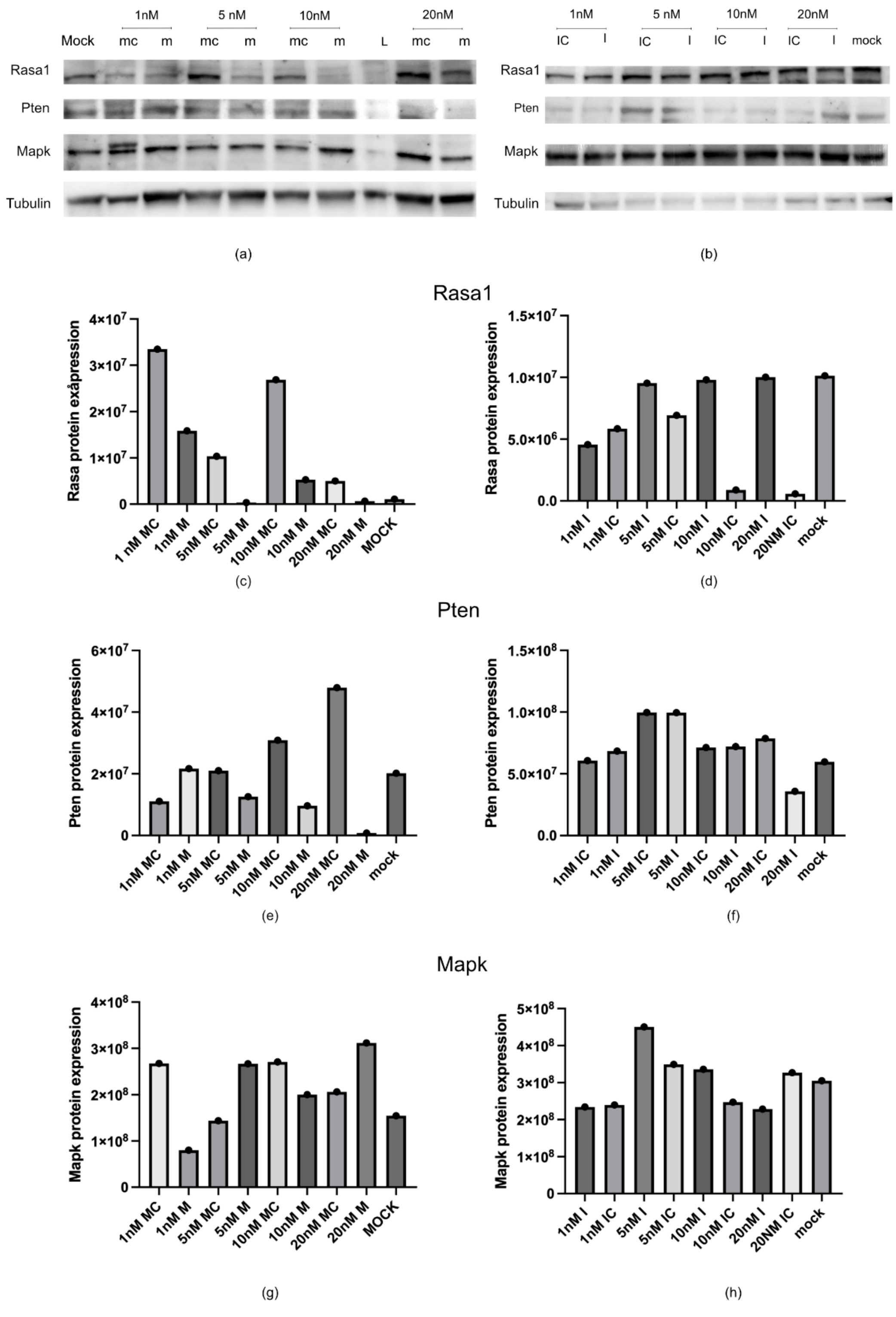

Supplementary Figure S1.

Representative image showing one of total two performed experiments analysing protein expression by Western blot visualizing Rasa, Pten and Mapk from primary urothelial cells upon transfection with transfection reagent alone (mock), or with specific double- and single stranded RNA molecules designed to mimic "M" (a) or inhibit "I" (b) endogenous miR-132 respectively and control for mimic and inhibitors molecules. Lower bars representing normalized values to the house keeping protein tubulin.
